# Redundant mechanisms regulating the proliferation vs. differentiation balance in the *C. elegans* germline

**DOI:** 10.3389/fcell.2022.960999

**Published:** 2022-09-02

**Authors:** Kara Vanden Broek, Xue Han, Dave Hansen

**Affiliations:** Department of Biological Sciences, University of Calgary, Calgary, AB, Canada

**Keywords:** stem cells, *C. elegans*, germline, germline stem cells, proliferation vs. differentiation balance, redundancy

## Abstract

The proper production of gametes over an extended portion of the life of an organism is essential for a high level of fitness. The balance between germline stem cell (GSC) proliferation (self-renewal) and differentiation (production of gametes) must be tightly regulated to ensure proper gamete production and overall fitness. Therefore, organisms have evolved robust regulatory systems to control this balance. Here we discuss the redundancy in the regulatory system that controls the proliferation vs. differentiation balance in the *C. elegans* hermaphrodite germline, and how this redundancy may contribute to robustness. We focus on the various types of redundancy utilized to regulate this balance, as well as the approaches that have enabled these redundant mechanisms to be uncovered.

## 1 Redundancy provides robust control

Proper development of multicellular organisms requires the ability to adapt to both internal and external perturbations. The ability of an organism to adapt to such changes requires its biological processes to be robust, meaning they are able to continue to develop and function, even when perturbations are encountered (Kitano, 2007; Wagner and Wright, 2007). This robustness allows for organisms to fine-tune their responses to varied conditions to ensure their proper development and reproductive success (Kafri et al., 2009). Multiple mechanisms are employed by an organism to allow for this robustness over a broad range of conditions. The focus of this review will be highlighting how redundancy can provide such robustness to an organism. Redundancy has been defined as “a situation in which there is an excess of causal components in a system, above the minimum needed for its proper function” (Láruson et al., 2020). In other words, redundancy in a system means that there is more than one way (gene, pathway, or process) to achieve the same overall outcome. To illustrate how redundancy can provide robustness to a system, as well as how redundancy can be identified, we provide an overview of some of the redundant mechanisms utilized within the *C. elegans* germline to ensure proper regulation of the balance between germline stem cell (GSC) proliferation and differentiation.

Stem cells are necessary for proper development and tissue homeostasis; therefore, the processes regulating their behavior must be robust. Stem cells have the ability of maintaining themselves over much of the life of the animal, while also providing the cells that form the differentiated tissue (Lin, 1997). For stem cells to function properly, there must be a tightly regulated balance between stem cell self-renewal (proliferation) and differentiation. If the processes regulating this balance are disrupted, development and tissue homeostasis will not occur properly. For example, excess self-renewal in the stem cell population will result in a tumor of proliferating stem cells and a reduction in the formation of differentiated cells or tissues [reviewed in (Fuchs and Chen, 2013)]. Conversely, if too many stem cells enter the pathway to differentiation, then the stem cell population could become depleted, resulting in an inability to form differentiated cells or tissues in the future. Therefore, the regulatory mechanisms that control the balance between stem cell proliferation and differentiation are highly robust, ensuring that a proper balance is maintained, even in many adverse conditions.

The reproductive success of animals within a population is key to that population being maintained; therefore, the processes involved in ensuring proper gamete formation must be robust. This includes the balance between proliferation and differentiation of GSCs. If this balance is disrupted, or is unable to withstand various environmental insults, the reproductive success of the animal would be significantly reduced; too much self-renewal would result in germline tumors of proliferating cells and a reduction or elimination of differentiated gametes that are formed, while too much differentiation would result in the GSC population being depleted and a lack of sustained gamete formation.

Genetic analyses of the proliferation vs. differentiation decision in model organisms has uncovered significant complexity, with many factors and cellular processes, contributing to the robustness of the systems [reviewed in (Lin, 1997; Singh and Hansen, 2017; Hubbard and Schedl, 2019)]. For example, analyses of the regulation of this balance in the *C. elegans* germline has revealed a core genetic pathway that contains many redundant factors, and many additional inputs that appear to fine-tune the balance. This regulatory mechanism has been extensively reviewed elsewhere (Hubbard and Schedl, 2019). In this review we will primarily focus on some of the diverse mechanisms of redundancy at play within the *C. elegans* germline to ensure optimal germline development and function.

**FIGURE 1:**
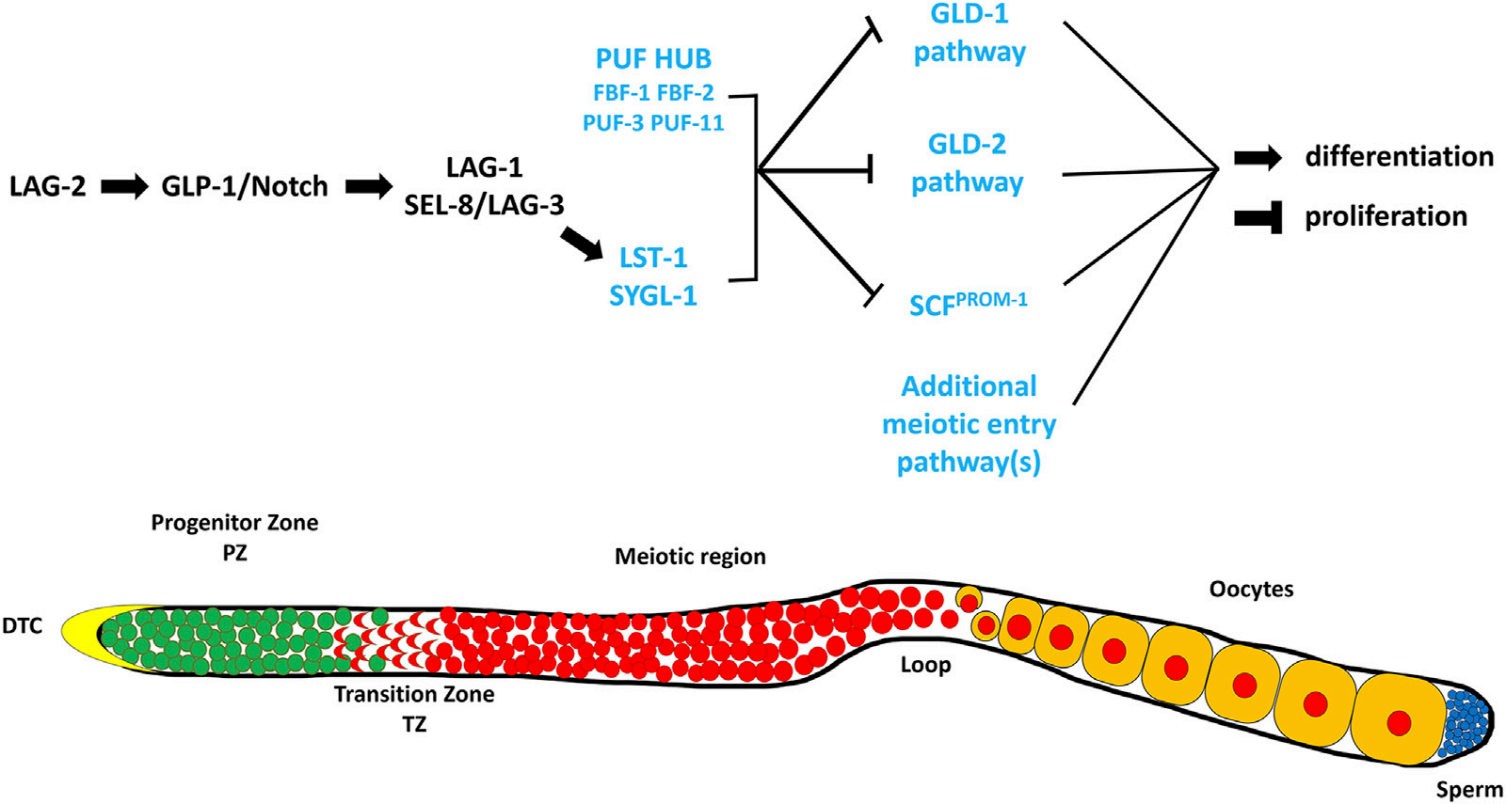
A simplified representation of the genetic pathway regulating the balance between GSC proliferation and differentiation in the germline of *C. elegans* (Adapted from (Hubbard and Schedl, 2019)). The DSL ligand, LAG-2, is expressed on the surface of the DTC where it interacts with the GLP-1/Notch receptor present on the membrane of the GSCs. This interaction results in the formation of a downstream transcriptional activation complex that activates the expression of LST-1 and SYGL-1. These two proteins work with components of the PUF HUB (FBF-1, FBF-2, PUF-3, and PUF-11) and promote proliferation and/or inhibit meiosis through repressing the downstream meiotic entry pathways, GLD-1 (GLD-1, NOS-3)*,* GLD-2 (GLD-2, GLD-3), SCF^PROM−1^ (SKR-2, CUL-1, PROM-1). The proteins and/or pathways whose elimination does not cause a major disruption in the proliferation vs. differentiation decision, except in a sensitized background, are labeled in blue. Below is a cartoon representation of a wildtype *C. elegans* hermaphrodite germline (Adapted from (Vanden Broek, 2021)). The distal end is on the left capped by the Distal Tip Cell (DTC). Green cells represent mitotic cells, red cells represent meiotic cells, blue cells represent mature sperm, and yellow cells represent developing oocytes.

The key signal that controls the proliferation vs. differentiation balance in the *C. elegans* germline emanates from the Distal Tip Cell (DTC), which is a somatic cell that caps the distal end of the gonad arm and serves as the niche cell for the GSC stem cell population (Kimble and White, 1981; Austin and Kimble, 1987; Kimble and Crittenden, 2007) (Figure 1). The DTC expresses ligands, including LAG-2 (Lin-12 and Glp-1 phenotype), which interact with the GLP-1/Notch (abnormal germ line proliferation) receptor on the surface of the GSCs (Henderson et al., 1994; Tax et al., 1997; Nadarajan et al., 2009; Greenwald and Kovall, 2013) (Figure 1). Upon ligand/receptor interaction the intracellular portion of GLP-1/Notch is thought to translocate to the nucleus and interact with the LAG-1 transcription factor and the SEL-8/LAG-3 transcriptional co-activator (Lambie et al., 1991; Christensen et al., 1996; Doyle et al., 2000; Petcherski and Kimble, 2000). This interaction results in the transcription of the *sygl-1* (synthetic germline proliferation defective) and *lst-1* (lateral signaling target) genes (Figure 1) (Kershner et al., 2014; Lee et al., 2016; Shin et al., 2017; Chen et al., 2020), whose protein products work with FBF-1, FBF-2 (*fem-3* mRNA binding factor) and other PUF (Pumilio and FBF) homologues (Crittenden et al., 2002; Shin et al., 2017; Haupt et al., 2019, Haupt et al., 2020; Wang et al., 2020). This PUF Hub inhibits the activities of at least three downstream genetic pathways that either inhibit proliferation and/or promote differentiation/meiotic entry (Figure 1). These pathways are referred to as the GLD-1, GLD-2 (defective in germ line development) and SCF^PROM−1^ (skp1-cullin-F-box protein progression of meiosis-1) pathways, based on their founding members (Kadyk and Kimble, 1998; Wang et al., 2002; Hansen et al., 2004a; Eckmann et al., 2004; Mohammad et al., 2018).

**FIGURE 2:**
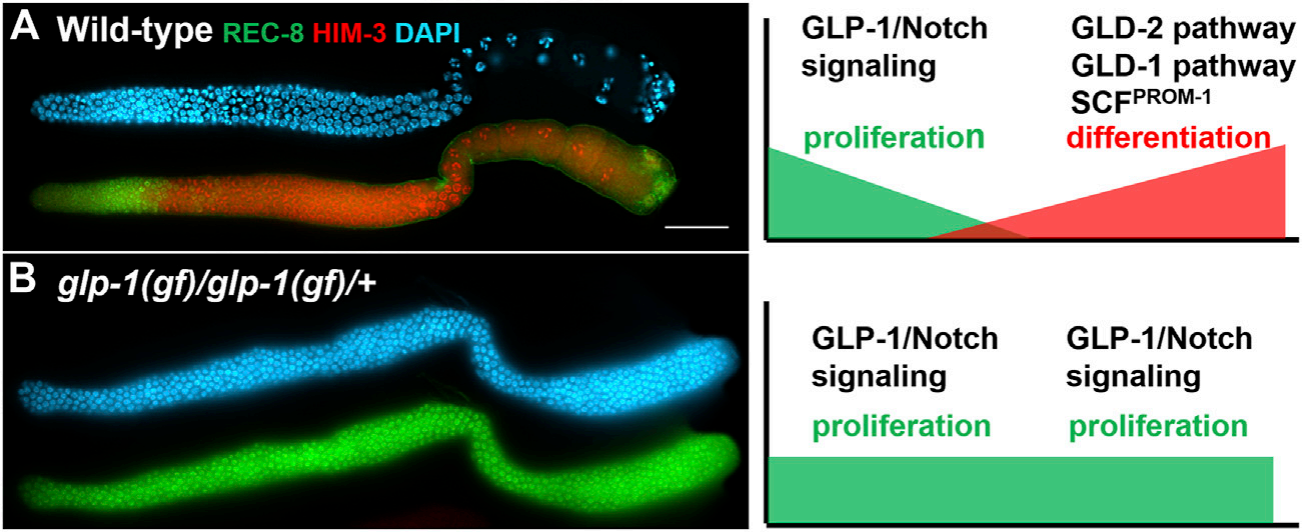
Dissected adult hermaphrodite gonad arms stained with a proliferative marker (green: anti-REC-8), a meiotic marker (red: anti-HIM-3), and DAPI to visualize DNA (blue). On the right are models depicting relative levels of GLP-1/Notch signaling and the GLD-1, GLD-2 and SCF^PROM−1^ downstream pathways **(A)** Wild-type adult hermaphrodite (adapted from (Hansen et al., 2004a)). **(B)** A tumorous germline from *glp-1(oz112gf)* homozygous hermaphrodite that also carries a wild-type copy of *glp-1* on a free duplication (adapted from (Hansen et al., 2004b)). Scale bar: 20 micron.

In the very distal end of the gonad, close to the DTC, GLP-1/Notch signaling levels are high, keeping cells in their proliferative or self-renewing state (reviewed in (Kimble and Simpson, 1997; Kimble and Crittenden, 2007) (Figure 2). As cells in the distal end divide, some cells progress more proximally, away from the DTC, resulting in a decrease in GLP-1/Notch signaling levels, allowing for the activities of the GLD-1, GLD-2 and SCF^PROM−1^ pathways to increase ((Kadyk and Kimble, 1998; Wang et al., 2002; Hansen et al., 2004a; Eckmann et al., 2004; Mohammad et al., 2018) reviewed in (Hubbard and Schedl, 2019)) (Figure 2). The increase in the activities of these pathways results in cells ceasing to proliferate/self-renew, but rather beginning to differentiate. This basic model of how the proliferation vs differentiation decision is controlled, including canonical Notch signaling inhibiting the activities of three downstream pathways, is overlaid by many additional factors and processes that presumably finetune this regulatory pathway to provide robustness to the system. Here, we focus on the factors and processes that contribute to this regulation but appear to do so with at least some level of redundancy. In other words, when their activities are reduced or eliminated in an otherwise wild-type genetic background, the balance between proliferation and differentiation is maintained; it is only when the activities of these factors are removed in combination with the removal of other factors, or in other sensitized backgrounds, that a severe disruption in the proliferation vs. differentiation balance is observed. These factors can be considered as functioning redundantly to regulate this balance in the *C. elegans* germline.

## 2 Redundancy can occur through different mechanisms

As mentioned above, redundancy refers to having more components in a system than what is necessary for proper function. With respect to the proliferation vs. differentiation balance in the *C. elegans* germline, redundancy can be inferred when components involved in regulating this balance can be removed, yet the proliferation vs. differentiation balance is maintained. For our discussion of redundancy within the *C. elegans* germline, we will characterize redundancy following Ghosh and O’Connor’s definitions (Ghosh and O’Connor, 2017). Their description of redundancy focuses on five types of redundancy that can occur within an organism—molecular redundancy, target redundancy, pathway redundancy, cellular process redundancy and system redundancy (Ghosh and O’Connor, 2017). Our discussion of the *C. elegans* germline will focus on the first four types of redundancy.

Molecular redundancy is when two or more effectors utilize the same molecular mechanism to act on or regulate the same target (Ghosh and O’Connor, 2017). When two effectors are molecularly redundant, they are able to completely compensate for the loss of the other as they provide the same function via the same biological activity. In order to be able to perform the same action, molecularly redundant genes often arise due to gene duplications with minimal changes to the gene sequence (Ghosh and O’Connor, 2017).

Target redundancy is when the target protein is modulated by two or more effectors, utilizing different mechanisms (Ghosh and O’Connor, 2017). In this type of redundancy, the two or more effectors will have the same overall impact on the target protein but with each effector utilizing a unique mechanism to do so. Therefore, each effector will be unable to completely replace the other effector(s) with respect to their molecular mechanisms, but the same intended impact on the target will occur, even if one effector is absent. In contrast to molecularly redundant genes, genes that have target redundancy may not share any sequence similarities (DNA or protein) (Ghosh and O’Connor, 2017).

Pathway redundancy is when effectors have different targets, but these targets are part of a single pathway that regulates the same overall process (Ghosh and O’Connor, 2017). When two or more effectors demonstrate pathway redundancy, loss of either single effector does not impact the intended outcome of the pathway; however, loss of both will result in the failure of the intended pathway outcome.

Cellular process redundancy is when effectors regulate redundant or complementary pathways that together control a cellular process; therefore, the cellular process is still able to occur if one of the redundant pathways is no longer functioning (Ghosh and O’Connor, 2017). When two effectors demonstrate cellular process redundancy they will function, through different mechanisms, targets, or pathways, to promote the same cellular process/outcome. Since they function towards the same outcome, the absence of a single effector does not prevent the cellular process from occurring; however, loss of two or more effectors will result in the failure of that cellular process to occur, as it is no longer promoted.

Identifying and understanding the type of redundancy regulating a process requires not just analysis of the overall outcome (phenotype) of a loss of the redundant effectors, but also the mechanism by which each effector functions. With many of the examples of redundancy in regulating the proliferation vs differentiation balance in the *C. elegans* germline, our understanding of the targets and biochemical functions of the effectors is not complete, making classification difficult in some instances; however, it is evident that many different types of redundancy are utilized to maintain this balance. We suggest that these different types of redundancy help ensure that this balance is maintained, even in varied environmental conditions, such as different temperatures, access to food/nutrition, crowding, etc.

## 3 The *C. elegans* germline is a powerful system to uncover redundancy

**FIGURE 3:**
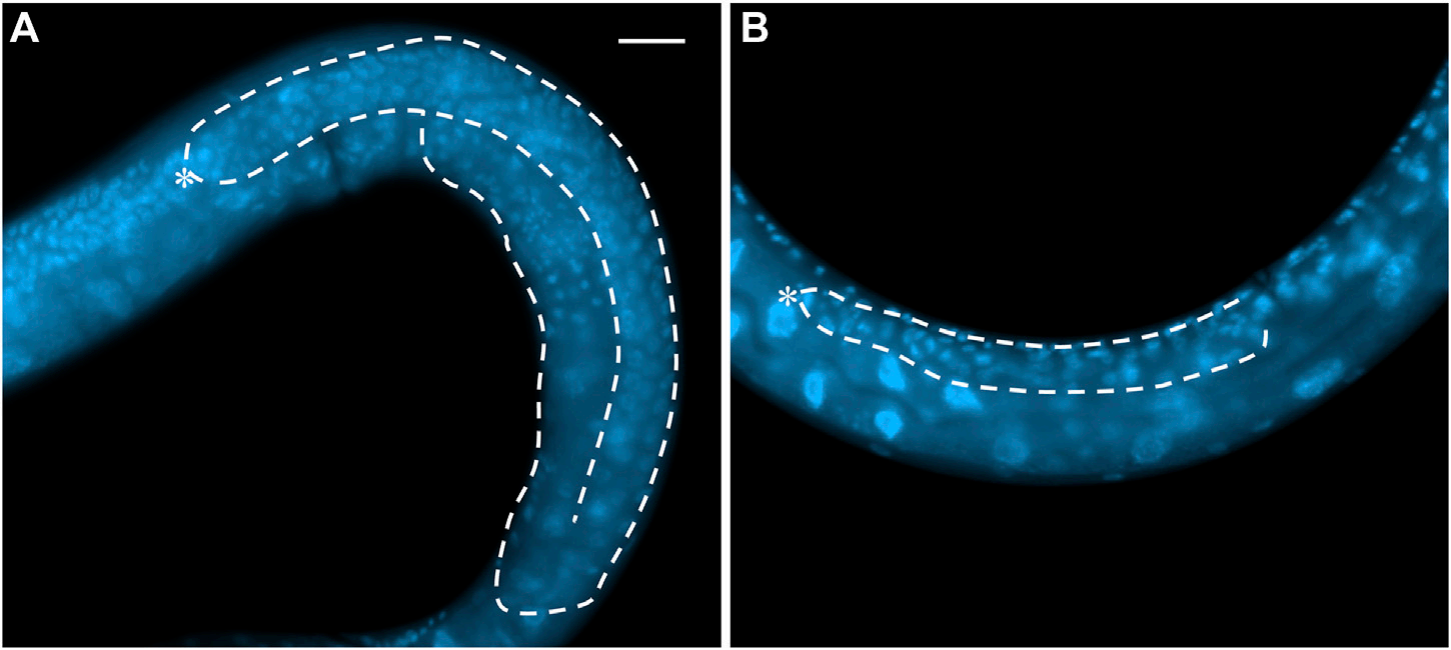
Whole mount DAPI staining of **(A)** an L4 wild-type hermaphrodite and **(B)** a GLP-1/Notch signaling mutant (actual genotype *glp-1(bn18ts)* grown at 25°C). White dash line shows the outline of one gonad arm. Asterisk: DTC. Scale bar: 20 µm.

The control of the balance between proliferation and differentiation in the *C. elegans* germline has proven to be a powerful system to uncover redundant factors and mechanisms due, in part, to the availability of sensitive genetic backgrounds and the ease with which subtle phenotypes can be observed. *C. elegans* are transparent, allowing observation of the germline in living animals [reviewed in (Corsi et al., 2015)]. The stem cells reside at the distal end of the gonad arm in a region referred to as the progenitor zone. Also within the progenitor zone are cells that are completing their final mitotic cell cycle prior to entering meiosis (progenitor cells), and cells that are in meiotic S phase [reviewed in (Hubbard and Schedl, 2019)]. The amount of proliferation occurring in the progenitor zone allows for the number of cells in this zone (∼200–250 cells) to remain relatively constant throughout adulthood, even as cells exit this zone by entering meiotic prophase. Cells enter meiotic prophase as they progress proximally down the gonad arm, eventually differentiating as gametes. In the hermaphrodite, sperm are first produced, during the late larval stages, then all subsequent gametes are oocytes, which are produced throughout most of adulthood (Hubbard and Greenstein, 2005). The oocytes grow as they progress down the gonad arm and take in yolk, which causes them to have a more yellow and granular appearance than the more clear proliferative cells in the distal end (Greenstein, 2005). Therefore, using a standard dissecting microscope, one can distinguish between regions of the gonad containing proliferative cells, and those containing oocytes. Mutant animals whose gonads consist of only proliferative cells can easily be identified using a dissecting microscope as their gonads appear much clearer due to the lack of oocytes that accumulate yolk. The degree of over-proliferation (more mitotically dividing cells than in a wild-type gonad) can vary depending on the mutation. Some mutants have only a modest increase in the amount of over-proliferation, resulting in a modestly larger progenitor zone, while other mutants have much more over-proliferation, resulting in gonads full of mitotically dividing cells and no differentiating cells. In animals with a high degree of over-proliferation, the gonads can appear enlarged, and are often referred to as germline tumors (Berry et al., 1997; Kadyk and Kimble, 1998; Hansen et al., 2004b). Conversely, mutant animals in which the stem cells prematurely differentiate, depleting the stem cell population very early in larval development, results in the gonad arms being largely devoid of germ cells, and the gonad being small and deflated (Kimble and White, 1981; Austin and Kimble, 1987). Under the dissecting microscope these mutants appear to lack extended gonad arms. These two extreme phenotypes, over-proliferation with a complete lack of differentiated cells, and early larval premature differentiation of the stem cells, are observed with gain-of-function (over-proliferation) or null alleles (premature differentiation) of the *glp-1* gene, which encodes a homologue of the Notch receptor. Animals homozygous for the strong *glp-1(oz112)* gain-of-function allele, perhaps the strongest identified *glp-1* gain-of-function allele, and which also contain an additional wild-type copy of *glp-1* on a free duplication, have completely tumorous germlines with no evidence of cells entering meiosis (Berry et al., 1997) (Figure 2). Conversely, in animals homozygous for a null allele for *glp-1*, such as *glp-1(q175)*, all stem cells differentiate prematurely in early larval development, resulting in a total of approximately four to eight stem cells, which differentiate into 16–32 sperm (Austin and Kimble, 1987; Kodoyianni et al., 1992) (Figure 3). The loss of the stem cell population is referred to as a Glp phenotype. Different mutations can cause the depletion of the stem cell population to occur at different stages of development; therefore, the total number of gametes produced can vary between mutants.

While *glp-1(oz112gf)* and *glp-1(q175null)* result in these extreme opposite phenotypes, there are many other alleles of *glp-1*, including weak gain-of-function alleles, and partial loss-of-function alleles, which result in milder phenotypes, especially at permissive temperatures (Austin and Kimble, 1987; Kodoyianni et al., 1992; Kerins et al., 2010). For example, *glp-1(oz264gf)* or *glp-1(ar202gf)* at higher temperatures result in increased proliferation of the mitotic cells and a delay of entry into the differentiation pathway, resulting in a larger than wild-type progenitor zone (Pepper et al., 2003; Kerins et al., 2010). At lower temperatures the amount of stem cell proliferation is much more similar to wild-type (Pepper et al., 2003; Kerins et al., 2010). Conversely, partial loss-of-function alleles of *glp-1*, such as *glp-1(bn18lf)*, have stem cells entering the differentiation pathway more distally in the gonad arm, resulting in a smaller progenitor zone than wild-type (Kodoyianni et al., 1992). At higher temperatures, all stem cells eventually differentiate, resulting in a depletion of the stem cell population; however, at lower temperatures the stem cell population is maintained. These weaker *glp-1* gain-of-function and loss-of-function alleles have been extremely useful in identifying other factors that are involved in regulating the proliferation vs. differentiation balance, as well as in testing other genes for involvement in this regulation. For example, genetic screens have been performed to identify mutations that either enhance or suppress the Glp or tumorous phenotypes produced by partial loss-of-function and gain-of-function alleles of *glp-1* (Kodoyianni et al., 1992; Maine and Kimble, 1993; Mantina et al., 2009; Racher and Hansen, 2012; Wang et al., 2012; Safdar et al., 2016). Therefore, these loss and gain-of-function alleles are utilized as sensitized genetic backgrounds. The screens are often performed at temperatures such that the *glp-1* allele on its own results in a mild or no mutant phenotype, and only when another mutant is present, will a Glp or tumorous phenotype occur. Additionally, many of the mutants identified do not cause a disruption in the proliferation vs. differentiation balance on their own; it is only when in a sensitized genetic background that a Glp or tumorous phenotype results. Therefore, the sensitivity of these genetic backgrounds, combined with the ease by which Glp and tumorous phenotypes can be observed in living animals, has resulted in the identification of many factors involved in regulating the proliferation vs. differentiation balance. Since loss of the activities of these factors, on their own, does not produce a proliferation vs. differentiation phenotype, they may be acting redundantly in regulating this process. Additionally, there have also been many factors identified through other means (e.g., homology, other genetic screens, physical interaction to known regulators, etc.) as potentially being involved in regulating the proliferation vs. differentiation balance whose involvement have been characterized by analyzing the Glp and tumorous phenotypes that result when combined with weak *glp-1* loss and gain-of-function alleles [Reviewed in (Hubbard and Schedl, 2019)]. Since mutations in many of these factors do not have a proliferation vs. differentiation phenotype on their own, some of these mutations have also been utilized as sensitized genetic backgrounds for genetic screens and epistatic analyses, which has helped to uncover additional redundant factors and processes [Reviewed in (Hubbard and Schedl, 2019)].

## 4 Examples of redundancy in regulating the proliferation vs differentiation balance in the *C. elegans* hermaphrodite

### 4.1 FBF-1 and FBF-2

*fbf-1* and *fbf-2,* referred to collectively as *fbf* (*fem-3* binding factor)*,* function downstream of GLP-1/Notch signaling within the *C. elegans* germline (Crittenden et al., 2002; Lamont et al., 2004). They share 93% identity in nucleotide sequence within their coding region and 91% identity at the amino acid level (Zhang et al., 1997). This high degree of similarity suggests they arose from a relatively recent duplication event (Crittenden et al., 2002; Wickens et al., 2002; Stumpf et al., 2008). The FBFs are homologous to Pumilio, a known RNA-binding protein in *Drosophila* (Murata and Wharton, 1995; Zhang et al., 1997). Key to PUF family proteins is the presence of a domain of eight PUF repeats that allows the proteins to specifically recognize and bind to conserved sequences present within target mRNAs (Zhang et al., 1997; Lamont et al., 2004; Bernstein et al., 2005). Within these PUF repeats FBF-1 and FBF-2 are 95% identical, differing by only 1 amino acid in this RNA-binding domain (Zhang et al., 1997; Wang et al., 2020). The similarity between FBF-1 and FBF-2 results in them binding and regulating the same mRNA targets through the conserved FBF-response element (FBE) (Koh et al., 2009; Kershner and Kimble, 2010; Prasad et al., 2016; Porter et al., 2019).

*fbf-1* and *fbf-2* were first characterized for their role in regulating the sperm to oocyte transition within the hermaphrodite germline through repressing the expression of FEM-3 (Zhang et al., 1997). A reduction in *fbf* expression, by RNAi, resulted in masculinization of the germline (excess sperm production, with abnormal or no oocytes produced) (Zhang et al., 1997). The high levels of similarity between *fbf-1* and *fbf-2* made it difficult to tease apart their specific roles; however, RNAi knockdown data, which due to the similarity between *fbf-1* and *fbf-2* likely reduced the function of both genes simultaneously, suggested that these genes are likely redundant for their role in the sex determination pathway and regulation of *fem-3* (Zhang et al., 1997). No single mutation in *fbf-1* or *fbf-2* was identified in genetic screens designed to identify mutants phenocopying *fbf* RNAi (Zhang et al., 1997), supporting the idea the *fbf-1* and *fbf-2* function redundantly to regulate sex determination within the GSCs.

Analysis of genetic null alleles of *fbf-1* and *fbf-2* identified an additional role for *fbf* in regulating the balance between proliferation and differentiation of GSCs (Crittenden et al., 2002). Loss of both *fbf-1* and *fbf-*2 resulted in a failure to initiate oogenesis, resulting in masculinization of the germline (Crittenden et al., 2002). In addition, the double mutant germlines lacked the progenitor zone, including GSCs, with all cells within the germline having entered meiosis during the L4 stage (Crittenden et al., 2002). This Glp phenotype was not as severe as that observed with *glp-1(null)* mutants as the progenitor zone did not become fully depleted until later in larval development, and depletion did not fully occur at higher temperatures (Crittenden et al., 2002; Hansen et al., 2004a). Loss of *fbf-1* or *fbf-2* alone resulted in relatively wild-type germlines, with the presence of a distal mitotic GSC pool and developing oocytes, suggesting that similar to their role in sex determination, *fbf-1* and *fbf-2* function redundantly to regulate the GSC proliferation vs. differentiation balance (Crittenden et al., 2002; Lamont et al., 2004).

These genes appear to function redundantly within the germline; however, detailed phenotypic analyses have uncovered independent roles, and different means of inhibition upon binding to the RNA target, for *fbf-1* and *fbf-2.* While both single mutants possess a GSC pool, *fbf-1* mutants have a reduction in the size of the progenitor zone, where *fbf-2* mutants have a larger than normal progenitor zone (Lamont et al., 2004; Wang et al., 2020). Cell cycle analysis in these mutants determined that *fbf-2* is required to regulate meiotic entry, with a loss of *fbf-2* resulting in a reduced rate of meiotic entry and decreased cell division rates leading to an increase in the progenitor zone (Wang et al., 2020). Conversely, *fbf-1* is required to prevent meiotic entry and control cell cycle progression, with a loss of *fbf-1* resulting in an increased rate of meiotic entry leading to a decrease in the progenitor zone (Wang et al., 2020).

Consistent with the idea that *fbf-1* and *fbf-2* have distinct roles and mechanisms of action within the germline is the difference in their expression pattern and subcellular localization. FBF-1 is expressed at high levels throughout the progenitor zone in both cytoplasmic and perinuclear foci (Crittenden et al., 2002; Lamont et al., 2004; Wang et al., 2020), whereas FBF-2 expression is detected ∼5 cell diameters from the distal most end of the gonad and throughout the progenitor zone in primarily perinuclear foci (Lamont et al., 2004; Voronina et al., 2012). FBF-1 co-localizes with CCF-1 (*C. elegans Caf1*), a catalytic subunit of the CCR4-NOT (carbon catabolite repressor factor 4—negative on TATA-less) deadenylation complex (Wang et al., 2020). Moreover, the interaction between FBF-1 and CCR4-NOT deadenylation complex appears to be required for FBF-1 mediated translational repression (Wang et al., 2020). FBF-2 localizes to P granules, and this localization is lost in *pgl-1* mutants (p-granule abnormality 1) (Voronina et al., 2012). Localization at P granules appears to be required for optimal FBF-2 target repression by allowing FBF-2 to form ribonucleoprotein complexes with its target mRNAs and repress them in a non-deadenylation manner (Voronina et al., 2012; Wang et al., 2020). Therefore, it is thought that FBF-1 represses targets through mRNA deadenylation and reduction in mRNA levels, whereas FBF-2 silences targets by inhibiting mRNA translation (Voronina et al., 2012; Wang et al., 2020). Although highly conserved within their PUF domains, FBF-1 and FBF-2 show only 87% and 72% identity in their N- and C-termini, respectively (Wang et al., 2020). These variable regions were recently shown to provide the specificity of binding partners for FBF-1 and FBF-2 [See (Wang et al., 2020) for discussion], and control their distinct localization patterns with the germline (Wang et al., 2020).

Initial analysis of *fbf-1* and *fbf-2* within the *C. elegans* germline suggested that these genes displayed molecular redundancy, with their highly conserved PUF domains allowing them to recognize and repress the same mRNA targets; however, more recent evidence of differing binding partners and localization, combined with their single mutant phenotypes, has uncovered that these two genes likely function using distinct mechanisms to repress target gene expression (Crittenden et al., 2002; Lamont et al., 2004; Voronina et al., 2012; Wang et al., 2020); therefore, *fbf-1* and *fbf-2* appear to display target redundancy in order to regulate both the proliferation vs differentiation balance and sex determination within the germline (Table 1).

**TABLE 1:** Examples of redundancy within the *C. elegans* proliferation versus differentiation decision.

Type of redundancy	Description	Germline Example
Molecular	Effectors function through identical mechanisms to regulate the exact target	PUF-3 **↔** PUF-11^a^
		LST-1 **↔** SYGL-1^b^
Target	Effectors modulate the same target but through unique mechanisms	FBF-1 **↔** FBF-2
		PUF-3 **↔** PUF-11^a^
		FBF-1 **↔** FBF-2 **↔** PUF-3 **↔** PUF-11^c^
		LST-1 **↔** SYGL-1^b^
Pathway	Effectors regulate different targets within the same overall pathway	FBF-1 **↔** FBF-2 **↔** PUF-3 **↔** PUF-11^c^
Cellular	Effectors regulate complementary pathways which control the same cellular outcome	GLD-1 **↔** GLD-2 **↔** SCF^PROM−1^
		Splicing factors **↔** GLD-2
		Protein degradation **↔** GLD-2

a Based on the current data PUF-3 and PUF-11 could utilize different types of redundancy. See section 4.2 for full explanation.

b Based on the current data LST-1 and SYGL-1 could utilize different types of redundancy. See section 4.3 for full explanation

c Based on the Current data the PUF hub components (FBF-1, FBF-2, PUF-11, PUF-3) could utilize different types of redundancy. See section 4.2 for full explanation.

### 4.2 The PUF Hub

Although the complete loss of the GSC pool in adult *fbf-1 fbf-2* double mutants highlights that these two genes function redundantly to promote GSC proliferation, the loss of the GSC pool was reminiscent, but still distinct, from that of a loss of GLP-1/Notch signaling. Loss of GLP-1/Notch signaling, through *glp-1* null mutants, results in four to eight GSCs that prematurely differentiate in early larval stages resulting in only 16–32 mature sperm (Glp phenotype) (Kimble and White, 1981; Austin and Kimble, 1987; Lambie et al., 1991). In the *fbf-1 fbf-2* double mutants the GSCs proliferate relatively normally until the L4 stage when they prematurely enter meiosis, resulting in a germline filled with mature sperm (∼400 sperm/animal) (Crittenden et al., 2002). The inability of *fbf-1 fbf-2* to completely phenocopy the loss of GLP-1/Notch signaling suggests that an additional component(s) works redundantly with *fbf*s to robustly promote GSC proliferation. PUF-11 was identified as a strong candidate as it displays similar RNA-binding specificity to FBF and interacts with LST-1 in a large scale yeast two-hybrid screen (Bernstein et al., 2005; Boxem et al., 2008; Koh et al., 2009; Haupt et al., 2020). PUF-3, a PUF-11 paralog, was also identified as a strong candidate due to having a nearly identical amino acid sequence to PUF-11 (Haupt et al., 2020).

*puf-11* and *puf-3,* have ∼90% nucleotide identity with each other, and 88% identity at the amino acid level (Hubstenberger et al., 2012; Haupt et al., 2020; Spike et al., 2022); however, across their eight PUF repeats, PUF-11 and PUF-3, differ by only a single amino acid from each other [(Haupt et al., 2020) see Supplementary Figure S2]. The high degree of similarity within these repeats suggests that PUF-11 and PUF-3, like FBFs, likely recognize and bind to the same mRNA motif within their targets. Interestingly, PUF-11 has flexibility in target recognition through binding to three recognition elements (Koh et al., 2009). PUF-11 and PUF-3 are localized to cytoplasmic and perinuclear granules in the distal end of the germlines, and are also expressed in developing oocytes, with PUF-11 being expressed at higher levels (Haupt et al., 2020). The identity of these granules, and their other components, are currently unknown. Additionally, it is unclear what mechanism PUF-11 and PUF-3 utilize to regulate their targets.

*puf-3* and *puf-11* single mutants, as well as the double mutant, have relatively wild-type progenitor zone sizes, with the double mutant being defective for oogenesis (Hubstenberger et al., 2012; Haupt et al., 2020). However, loss of *puf-11* or *puf-3* in an *fbf-1 fbf-2* background results in a reduction in GSC proliferation as compared to *fbf-1 fbf-2* double mutants (Haupt et al., 2020). A loss of all four PUF genes in the quadruple mutant, *fbf-1 fbf-2; puf-3 puf-11,* results in a Glp phenotype virtually identical to that observed in *glp-1(null)* mutants (Haupt et al., 2020). These four redundant PUF proteins that regulate the GSC proliferation vs. differentiation balance, downstream of GLP-1/Notch signaling, are referred to as the PUF hub (Haupt et al., 2020). It is still unclear if, similar to FBF-1 and FBF-2*,* differences exist in the roles and mechanisms utilized by the PUF-11 and PUF-3 paralogs to regulate their target mRNAs. Understanding more about how exactly PUF-11 and PUF-3 function will help determine if these two proteins display molecular redundancy, or target redundancy, with each other as well as with FBF-1 & 2. Moreover, the similarities between the mRNA recognition motifs between PUF-3/-11 and FBF-1/-2, suggests the possibility that all four PUFs potentially target and control the same mRNAs. If true, this would suggest that all four PUFs provide target redundancy to ensure robustness to regulating the GSC proliferation vs differentiation balance within the germline (Table 1). However, the differences in the binding motif, and the flexibility displayed by PUF-11, suggest that the PUF proteins may repress distinct pools of mRNAs in order to ensure proper balance, thereby displaying pathway redundancy in order achieve the same overall outcome (i.e., inhibiting meiosis and/or sex determination) (Koh et al., 2009). It is entirely possible that the PUF hub components could be displaying both target and pathway redundancy to tightly control the GSC proliferation vs differentiation decision (Table 1).

### 4.3 LST-1 and SYGL-1

As mentioned above, ligand binding to the GLP-1/Notch receptor is thought to result in translocation of the intracellular portion of GLP-1 to the nucleus where it binds to the LAG-1 transcription factor, resulting in transcription of downstream genes (Kodoyianni et al., 1992; Crittenden et al., 1994; Greenwald and Kovall, 2013; Shaffer and Greenwald, 2022). However, it has been difficult to identify these transcriptional targets that regulate the GSC proliferation vs differentiation balance. The difficulty in identifying these target genes through various genetic screens suggested that these targets may not have strong proliferation vs differentiation phenotypes when they are individually mutated, but rather that they may act redundantly to promote the balance between proliferation and differentiation (Maine and Kimble, 1993; Wang et al., 2012; Kershner et al., 2014; Chen et al., 2020). Bioinformatic analysis identified a list of potential target genes based on the presence of a cluster of LAG-1 binding sites in their promoter regions, as well as their identification as proteins that bind to the FBFs [See (Kershner et al., 2014) for further explanation]. This list was further reduced to a single candidate, *sygl-1* (synthetic Glp, T27F6.4), based on mRNA *in situ* data showing expression within the GSC pool (Kershner et al., 2014). Following the hypothesis that GLP-1/Notch target genes most likely act redundantly, double RNAi with *sygl-1* and other candidate target genes were performed (Kershner et al., 2014). Only one combination, *sygl-1* and *lst-1* (lateral signaling target 1) knockdown, resulted in a Glp phenotype during larval development (Kershner et al., 2014). This Glp phenotype was verified using a *lst-1 sygl-1* double mutant and found to phenocopy a loss of *glp-1* (Kershner et al., 2014). Consistent with these two genes functioning redundantly to control the proliferation vs. differentiation balance, loss of *lst-1* or *sygl-1* alone results in relatively wild-type germlines with an intact progenitor zone (Kershner et al., 2014). *lst-1* and *sygl-1* are also required to promote GSC proliferation in adults, as reduction in both *lst-1* and *sgyl-1* in adults *via* double RNAi results in a loss of the GSC pool (Kershner et al., 2014). Genome-wide approaches found that *lst-1* and *sygl-1* are likely the only direct target genes of GLP-1/Notch signaling that regulate this balance (Chen et al., 2020).

Both *lst-1* and *sygl-1* appear to be conserved only within the *Caenorhabditis* genus (Kershner et al., 2014). Furthermore, LST-1 and SYGL-1 share little to no sequence similarity with each other (Kershner et al., 2014). SYGL-1 has no predicted motifs or domains whereas LST-1 has a single predicted nanos-like zinc finger domain (Kershner et al., 2014); however, this domain is not required for LST-1’s ability to promote GSC proliferation, and instead depends on two KXXL FBF-binding motifs (Kershner et al., 2014; Haupt et al., 2019). Consistent with a role in regulating the GSC proliferation vs differentiation decision, SYGL-1 is expressed throughout the progenitor zone up to 15 germ cell diameters (gcd) away from the distal most end, whereas LST-1’s expression is restricted to the first five gcds (Lee et al., 2016).

The progenitor zone is slightly reduced, as compared to wild-type, in *lst-1* single mutants; however, loss of *sygl-1* results in a progenitor zone about half the size as that found in wild-type animals (Kershner et al., 2014; Brenner and Schedl, 2016; Shin et al., 2017). Interestingly, the smaller progenitor zone in *sygl-1* mutants corresponds to the region where LST-1 is expressed, suggesting that SYGL-1 expression sets up the GSC pool size, and in its absence LST-1 determines the pool size (Shin et al., 2017). This highlights the possibility that LST-1 and SYGL-1 may regulate the proliferation vs differentiation balance independently of each other, and potentially through distinct mechanisms. Ubiquitous expression of either LST-1 or SYGL-1 throughout the germline cells results in germline tumors, demonstrating that LST-1 and SYGL-1 alone are sufficient to promote GSC proliferation (Shin et al., 2017). Ubiquitous expression of these proteins is unable to rescue the loss of GSC proliferation in an *fbf-1 fbf-2* double mutant suggesting that LST-1 and SYGL-1 act with, or in parallel to, FBFs to function (Shin et al., 2017). Therefore, LST-1 and SYGL-1 may regulate the GSC proliferation vs differentiation balance through interacting with FBFs. This is supported by interactions between LST-1 and both FBF-1 and FBF-2 in a yeast 2-hybrid analysis (Shin et al., 2017; Haupt et al., 2019), and with the impact that LST-1 has on the RNA sequence bound by FBF-2 (Qiu et al., 2019). SYGL-1 interacts with both FBF-1 and FBF-2 as determined by yeast 2-hybrid analysis, with the interaction with FBF-2 being confirmed by immunoprecipitation (Shin et al., 2017). Furthermore, LST-1 and SYGL-1 were found to physically interact with PUF-11 and PUF-3, the other components of the PUF Hub, through yeast 2-hybrid analysis (Boxem et al., 2008; Haupt et al., 2020). The working model suggests that SYGL-1 and LST-1 function as FBF binding partners and are required for GSC maintenance through selective repression of target mRNAs (Shin et al., 2017; Haupt et al., 2020).

Further research will be required to determine if SYGL-1 and LST-1 are required to selectively target different FBF mRNA targets, or if they assist in regulating similar targets as is suggested based on the increase in GLD-1 levels in both *lst-1* and *sygl-1* mutants (Brenner and Schedl, 2016). An alternative interpretation is that SYGL-1 and LST-1 are required for optimal activity of PUF hub components, rather than target selectively. It is currently unclear what type of redundancy LST-1 and SYGL-1 display. If they both function to regulate the PUF hub components through the same mechanism and ultimately lead to the same outcome on FBF target gene expression then they would display molecularly redundancy. The lack of sequence similarity between SYGL-1 and LST-1, as well as differences in their expression domains, suggests that they may display target redundancy, regulating different components and/or actions of the PUF hub in order to promote GSC proliferation within the germline (Table 1).

### 4.4 Three redundant pathways inhibit proliferation and/or promote differentiation

Downstream of LST-1, SYGL-1 and the PUF Hub in regulating the proliferation vs differentiation balance in the *C. elegans* germline are three redundant pathways; GLD-1, GLD-2 and SCF^PROM−1^ (Kadyk and Kimble, 1998; Wang et al., 2002; Hansen et al., 2004a; Eckmann et al., 2004; Mohammad et al., 2018). If components of any one of these three pathways are eliminated, the balance between proliferation and differentiation occurs relatively normally; however, loss of genes in two or more pathways cause most GSCs to fail to differentiate/enter meiosis and results in a germline tumor of mostly undifferentiated cells. Therefore, none of the pathways are essential for the balance between GSC proliferation and differentiation to be maintained, suggesting that these pathways function redundantly.

#### 4.4.1 GLD-1 pathway

The GLD-1 pathway acts redundantly with the GLD-2 and SCF^PROM−1^ pathways to suppress proliferation and/or promote differentiation. In *gld-1* null single mutants, germs cells enter meiosis normally; however, in *gld-2 gld-1, gld-2 prom-1* or *gld-1 prom-1* double mutants germ cells fail to enter meiotic prophase (differentiate) normally and result in over-proliferation of the mitotic cells, with most cells failing to enter meiosis (Kadyk and Kimble, 1998; Hansen et al., 2004b; Mohammad et al., 2018). These double mutant germline over-proliferation phenotypes are epistatic to *glp-1*, suggesting that they function downstream of GLP-1/Notch signaling.

In the gonad GLD-1 localizes to the cytoplasm of the germ cells (Jones et al., 1996). GLD-1 protein levels are low in the distal region then increase gradually in more proximal cells that are entering into meiosis, with levels peaking in cells in the leptotene stage of meiosis prophase I; GLD-1 levels drop to background at the loop region (Jones et al., 1996; Brenner and Schedl, 2016). GLD-1 encodes a KH domain RNA-binding protein homologous to mammalian Quaking (Jones and Schedl, 1995; Lee and Schedl, 2010). GLD-1 is thought to function by binding to the 3′UTRs of target mRNAs and inhibiting their translation; however, the molecular mechanism by which GLD-1 represses mRNA activities is still largely unknown (Jan et al., 1999; Lee and Schedl, 2001; Marin and Evans, 2003; Biedermann et al., 2009; Jungkamp et al., 2011; Wright et al., 2011; Theil et al., 2019).

Within the GLD-1 pathway is the *Drosophila* Nanos ortholog, NOS-3 (nanos related). Its placement in this pathway is based on the finding that *gld-2; nos-3* double mutants show synthetic tumors while *gld-1; nos-3* double mutants have normal meiotic entry (Hansen et al., 2004a; Eckmann et al., 2004). NOS-3 is a cytoplasmic protein that is expressed throughout the germline (Kraemer et al., 1999). NOS-3 also functions alongside GLD-2 to regulate GLD-1 accumulation in the proximal progenitor zone, while the exact mechanism remains unclear (Hansen et al., 2004a; Brenner and Schedl, 2016).

#### 4.4.2 GLD-2 pathway

The core genes in the GLD-2 pathway are *gld-2* and *gld-3*. *gld-2* encodes a noncanonical poly-A polymerase (Kadyk and Kimble, 1998; Wang et al., 2002; Nousch et al., 2017), while *gld-3* encodes a Bicaudal-C family RNA-binding protein (Eckmann et al., 2002, 2004; Suh et al., 2006). Both GLD-2 and GLD-3 are expressed predominantly in the cytoplasm. In adult hermaphrodite germline, GLD-2 expression is low at the distal region, increases dramatically in the proximal region and oocytes, decreases during spermatogenesis and is excluded from mature sperm (Wang et al., 2002; Millonigg et al., 2014). The expression pattern of GLD-3 is very similar to that of GLD-2 (Eckmann et al., 2002). GLD-2 by itself has very low levels of poly-A polymerase activity; however its activity increases dramatically when bound to GLD-3 in *in vitro* assays (Wang et al., 2002). If the activity of any of the GLD-1 pathway genes are eliminated in combination with the activity of any of the GLD-2 pathway genes, a germline tumor results with most germ cells failing to enter meiosis (differentiate) (Hansen and Schedl, 2013). Therefore, the GLD-1 and GLD-2 pathways function redundantly to regulate the balance between GSC proliferation and differentiation [reviewed in (Hansen and Schedl, 2013)]. GLD-2 pathway is found to promote the activity of the GLD-1 pathway. GLD-2 and GLD-3 polyadenylate *gld-1* mRNA and enhance GLD-1 protein translation (Suh et al., 2006, 2009; Brenner and Schedl, 2016). Even though *gld-1* is identified as a target of GLD-2, the GLD-2 pathway must also act on other target genes to promote meiotic entry and/or inhibit proliferation, as germ cells enter meiosis normally in *gld-1* null single mutants but not in *gld-2 gld-1* double mutants [reviewed in (Hansen and Schedl, 2013)]. Since GLD-1 is thought to inhibit target gene activity by repressing translation, and GLD-2 is thought to promote target gene activity by polyadenylating mRNAs, a simple model is that the GLD-1 pathway inhibits genes that promote GSC proliferation, while the GLD-2 pathway promotes genes necessary for meiotic entry (differentiation); however, additional complexity is likely involved [reviewed in (Hansen and Schedl, 2013)]

#### 4.4.3 SCF^PROM−1^ acts redundantly with the GLD-1 and GLD-2 pathways

There is evidence of an additional pathway(s) that functions in parallel to the GLD-1 and GLD-2 pathways to inhibit proliferation and/or promote meiotic entry. First, the synthetic tumorous germlines in *gld-2 gld-1* double mutants are not completely tumorous, but rather contain some meiotic cells (Hansen et al., 2004b). Second, these meiotic cells are suppressed by a *glp-1* gain-of-function allele in the absence of the GLD-1 and GLD-2 pathways (*gld-2 gld-1; glp-1(gf)*) (Hansen et al., 2004a). Finally, knocking down the activity of the Cyclin E/CDK-2 gene *cye-1* in a *gld-2 gld-1*; *glp-1* triple mutant background results in widespread meiotic entry, especially in the distal-most region that normally shows no meiotic entry in *gld-2 gld-1* double mutants (Fox et al., 2011). Therefore, a third pathway likely acts in parallel to the GLD-1 and GLD-2 pathways, and can down regulate *cye-1* to promote meiotic entry (Fox et al., 2011).

A key player identified in this third pathway was the SCF (Skp1, Cullin, F-box) E3 ubiquitin-ligase complex which includes the PROM-1 F-box protein. SCF^PROM−1^ was found to bind CYE-1, potentially targeting it for degradation (Mohammad et al., 2018). Loss of *prom-1* in a *gld-1* or *gld-2* mutant background resulted in the formation of a synthetic tumor, indicating that PROM-1 acts in parallel with the GLD-1 and GLD-2 pathways (Mohammad et al., 2018). Furthermore, the number of meiotic cells was lower in *gld-2 gld-1 prom-1* pathway triple mutants as compared to *gld-2 gld-1* pathway double mutants (Mohammad et al., 2018). However, some meiotic cells were still present in the *gld-2 gld-1 prom-1* triple mutant, suggesting that there may be an additional pathway(s) that functions redundantly with the GLD-1, GLD-2 and SCF^PROM−1^ pathways to promote meiotic entry and/or inhibit proliferation (Mohammad et al., 2018).

The GLD-1, GLD-2 and SCF^PROM−1^ pathways display cellular process redundancy in their regulation of the proliferation vs differentiation decision in the *C. elegans* germline (Table 1). Each pathway is thought to function through different mechanisms to promote the same outcome, which is for germ cells to cease proliferating and enter meiosis. GLD-1 most likely acts by binding to the 3’ UTRs of genes that promote proliferation and inhibiting their translation; GLD-2 likely polyadenylates and stabilizes target mRNAs to allow for translation to occur, thereby allowing their protein products to promote meiotic entry; and SCF^PROM−1^ acts by directly and indirectly regulating four mitotic cell cycle proteins and a proposed protein that inhibits homolog pairing. Having multiple pathways acting through different mechanisms to regulate the switch from proliferation to differentiation may decrease the chance of a reduction in the activity of a specific cellular process affecting this switch.

### 4.5 Additional factors that disrupt the balance when their activity is removed in sensitized backgrounds

#### 4.5.1 Splicing factors

Many splicing factors have been identified as potentially being involved in the proliferation vs differentiation decision based on a reduction or loss of their activity enhancing tumor formation. For example, enhancer screens were performed designed to identify tumorous enhancers of weak *glp-1* gain-of-function alleles, from which *teg-1*, *teg-4* (tumorous enhancer of *glp-1(gf)*) and *prp-17* (Yeast PRP17 related splicing factor) were identified (Mantina et al., 2009; Kerins et al., 2010; Wang et al., 2012). TEG-1 is homologous to CD2BP2, which has been implicated in U4/U6.U5 tri-snRNP formation (Wang et al., 2012, 2017), TEG-4 is homologous to subunit 3 of SF3b (Mantina et al., 2009), and PRP-17 is homologous to the PRP17/CDC40 pre-mRNA splicing factor (Kerins et al., 2010). Other splicing factors have also been identified as being involved through other genetic screens or directly testing for potential involvement, including *mog-1, mog-4, mog-5* (masculinization of germline)*, cyn-4/mog-6* and *prp-19* (Graham et al., 1993; Graham and Kimble, 1993; Puoti and Kimble, 1999; Belfiore et al., 2004; Kerins et al., 2010; Gutnik et al., 2018). Importantly, reduction or loss of these factors alone does not result in a disruption of the proliferation vs. differentiation decision. It is only when these mutants are in a sensitized genetic background that a germline tumor results. Additionally, these splicing factors are involved in various steps of the splicing process, suggesting that it is not a disruption of a single splicing step that affects the proliferation vs. differentiation balance. It is currently unclear as to why a reduction in mRNA splicing would affect this balance. It is possible that there are one or more key factors involved in regulating this balance, and who are particularly sensitive to changes in splicing efficiency. It is also possible that splicing directly regulates the activity of a gene, potentially through alternative splicing, and that a reduction in splicing efficiency disrupts this regulation. However, with either of these or other possibilities, we currently do not know if one, a few, or many targets involved in regulating the proliferation vs. differentiation balance are misregulated when splicing efficiency is decreased.

Without knowing precisely why the loss of splicing efficiency disrupts the proliferation vs differentiation balance, or the downstream targets that are affected, it is difficult to categorize the type of redundancy they exhibit. However, since many of these splicing genes have been shown to likely function in the GLD-1 pathway due to their ability to form synthetic tumors with a loss of GLD-2 pathway genes, but not GLD-1 pathway genes (Belfiore et al., 2004; Mantina et al., 2009; Kerins et al., 2010; Wang et al., 2012), this suggests that the splicing factors likely function in the GLD-1 pathway; therefore, the redundancy observed between splicing factors and GLD-2 pathway genes is likely to be cellular process redundancy, as is likely the case with the GLD-1 and GLD-2 pathways (discussed above). Since the GLD-1 and GLD-2 pathways are likely to employ different mechanisms to regulate likely different targets, we consider this to be an example of cellular process redundancy (Table 1).

#### 4.5.2 Proteasomal degradation

In order to identify additional genes that could function in the GLD-1 pathway to regulate the proliferation vs. differentiation balance, a genetic screen was performed that identified mutations that result in a synthetic tumorous phenotype in *gld-2(null)* mutants (Hansen et al., 2004b). One of the mutations obtained in this screen was a partial loss-of-function allele of *pas-5* (proteasome alpha subunit), which encodes an ⍺-subunit of the 20S proteasome (Macdonald et al., 2008). It was found that a partial reduction of proteasomal activity results in an over-proliferation phenotype in sensitized backgrounds, suggesting that a proliferation promoting protein, or proteins, were not being properly degraded when proteasome activity was reduced. More than one proliferation promoting protein likely contributed to this over-proliferation as genetic analyses of the *pas-5* partial-loss-of-function allele revealed that it likely affected the Notch signaling pathway, as well as the GLD-1 pathway functioning downstream of GLP-1/Notch signaling (Macdonald et al., 2008). The chromodomain containing protein MRG-1 (homologous to mammalian MRG15; mortality-related gene) was found to be one of these proliferation promoting proteins, and is targeted for degradation by the E3 ubiquitin ligase RFP-1 (ring finger protein) (Gupta et al., 2015). Other factors that function in proteasomal degradation have been identified that result in over-proliferation when their function is lowered or removed in a *glp-1(gf)* background, suggesting that there could be multiple targets whose regulated degradation is necessary for the proliferation vs differentiation decision to be properly maintained (Pepper et al., 2003; Choi et al., 2010; Safdar et al., 2016). As mentioned, at least some of these targets likely function in the GLD-1 pathway since a reduction of proteasome activity is synthetic tumorous with the loss of GLD-2 pathway genes. Proteasomal degradation appears to be required to degrade proliferation promoting proteins, like MRG-1, to ultimately inhibit proliferation. Conversely, GLD-2 likely functions to promote the stability of mRNA targets required for differentiation. Since protein degradation and the GLD-2 pathway function through different mechanisms to regulate distinct targets and allow for differentiation to occur, these pathways most likely display cellular process redundancy (Table 1).

There are many other genes that have been identified as having a role in the proliferation vs. differentiation decision based, in part, on their ability to enhance the Glp phenotype of weak *glp-1* partial loss-of-function alleles, or enhance the tumorous phenotype of *glp-1* gain-of-function alleles [reviewed in (Hubbard and Schedl, 2019)]. The functions of the proteins encoded by these genes are diverse, including phosphorylation, members of an Argonaut complex, and RAS/MAP kinase signaling, as well as many others [reviewed in (Hubbard and Schedl, 2019)]. Since loss or reduction of the activities of these genes on their own does not result in a disruption of the proliferation vs. differentiation balance, but that a disruption of the balance does occur when their activities are reduced in a sensitized genetic background, we consider these factors as functioning redundantly in regulating this balance. The precise type of redundancy will require a detailed understanding of their biochemical function, genetic interactions with known components of the genetic pathway regulating this balance, and identification of the targets of their activity.

## 5 Conclusion

The genetic pathway regulating the proliferation vs. differentiation decision in the *C. elegans* germline contains considerable redundancy in that there are many factors that contribute to the control of this decision whose loss does not result in a severe disruption to the balance, except when in a genetic background that is sensitized to either over-proliferation or under-proliferation. While the redundancies we have discussed are primarily thought to act in the GSCs and their ability to remain GSCs or enter a pathway to differentiation, it is also possible that redundancies may exist in controlling the behavior of the other cells in the progenitor zone (those completing mitosis and those in meiotic S phase), which could impact over-proliferation and under-proliferation phenotypes. Additionally, the type and degree of redundancy may not be constant throughout development and ageing. For example, it is possible that some factors are redundant in early larval development as the progenitor zone is being established, but not in adulthood while it is being maintained.

Presumably, even slight disruptions in the proliferation vs. differentiation balance would negatively impact long-term reproductive success. Therefore, there is likely significant evolutionary pressure ensuring that the system regulating the proliferation vs differentiation balance is robust, which could involve a significant level of redundancy. It is thought that redundancy can provide robustness to a system by decreasing the phenotypic consequence of mutations, or by broadening the flexibility of the system so that signals can still be properly controlled in fluctuating cellular environments, different genetic backgrounds, and environmental stresses such as changes in temperature and availability of food (Kirschner and Gerhart, 2005; Kafri et al., 2009). Therefore, it is fitting that the process regulating the GSC proliferation vs differentiation decision, which is tightly linked to the reproductive success of the animal, would utilize redundancy to ensure that this decision is tightly controlled (Civetta et al., 2006; Bauer DuMont et al., 2007; Flores et al., 2015; Whittle and Extavour, 2019).

In this review we have discussed some of the different types of redundancy, highlighted with specific examples, that are utilized in regulating the proliferation vs differentiation decision in the *C. elegans* germline. In some cases, the initial discovery of redundant factors and their presence or lack of similarity suggested that they were an example of one class of redundancy. However, for some redundant factors, as understanding increased as to their modes of action and their potential targets, it actually became less clear as to which class of redundancy most accurately described their relationship. Furthermore, some redundant factors may exist in more than one class; for example, two redundant proteins with a high degree of amino acid sequence identity may function on a group of common targets, but may also have targets that are unique to each redundant factor. Conclusive classification of redundant factors requires complete understanding of the biochemical functions of the factors and a knowledge of all their targets. Obviously, we are not yet at that level of understanding with respect to the regulation of the proliferation vs differentiation decision in the *C. elegans* germline. However, perhaps our level of understanding of the biochemical functions and targets involved in this process is relatively high, which has revealed that the redundant relationships between factors are more complex than previously appreciated. Indeed, the relationships between redundant factors in other systems may also be found to be more complex than once thought as more is learned about the biochemical functions and targets of these redundant factors. However, what is clear is that many types of redundancy are utilized in regulating this proliferation vs. differentiation balance in the *C. elegans* germline, and that this redundancy likely contributes to its reproductive success and fitness.
